# Fungal and algal lichen symbionts show different transcriptional expression patterns in two climate zones

**DOI:** 10.1098/rspb.2024.2962

**Published:** 2025-07-02

**Authors:** Henrique Fernandes Valim, Jürgen Otte, Imke Schmitt

**Affiliations:** ^1^ Senckenberg Biodiversity and Climate Research Centre, Frankfurt, Hesse 60325, Germany; ^2^ Department of Biosciences, Goethe University Frankfurt, Frankfurt, Hesse 60438, Germany

**Keywords:** light induction, metatranscriptome, gene expression, symbiosis, algae, fungi

## Abstract

In the lichen symbiosis, the fungal and algal partners constitute a closely integrated system. The combination of fungal and algal partners changes along climate gradients in many species, and is expected to be adaptive. However, the functional mechanisms behind this symbiosis-mediated environmental adaptation are unknown. We investigated which transcriptional profiles are associated with specific fungal–algal symbiont pairings found in lichens from high-elevation (Lower Supratemperate) and low-elevation (Lower Mesomediterranean) sites at two extremes of a climatic gradient on Mount Limbara, Sardinia. Using laboratory-acclimatized thalli, we found that lichen fungal and algal symbionts show variable expression profiles between high- and low-elevation individuals: circadian- and temperature-associated genes for fungi and light-responsive genes for algae show climate-specific patterns. High- and low-elevation individuals differentially express sugar transporters in both symbionts, pointing to symmetrical and climate-dependent sugar transport mechanisms between them. A light pulse treatment identified asymmetries between fungal and algal light responses, with high- and low-elevation fungal symbionts but only low-elevation algal symbionts showing a response. Together, these results tie previously observed genomic variation along climatic gradients in a lichen species to functional differences in transcription for the fungal and algal symbionts, contributing to our understanding of environmental specialization and niche-specific partner combinations in lichens.

## 1. Background

Lichens are evolutionarily stable symbioses composed of a primary fungal partner, the mycobiont, and an algal or cyanobacterial partner, the photobiont [1–3]. Typically, lichens are understood as a mutualism wherein the photobiont provides a photosynthesis-derived carbohydrate source to the mycobiont, while the mycobiont provides micronutrients, a better hydration regime, protection against grazing and environmental protection for the photobiont (reviewed in [2]). Many lichen mycobionts have wide or near-cosmopolitan distributions [4] and associate with multiple photobiont species along their range, e.g. *Lecanora rupicola* [5], *Evernia mesomorpha* [6], *Thamnolia vermicularis* [7], *Cetraria aculeata* [8], *Tephromela atra* [9], *Ramalina menziesii* [10] and *Umbilicaria pustulata* [11].

Studies along geographic gradients have linked environmental factors to photobiont distribution patterns. In the genus *Cladonia*, 172 mycobiont species associate with 545 lineages of *Asterochloris* photobionts across a cosmopolitan range, with both climate and fungal identity driving genetic variation in the photobiont [12]. A narrower analysis of 1000 *Cladonia* spp. samples within Europe revealed four non-overlapping sets of myco- and photobiont lineages, each associated with specific ecological conditions, and with photobiont switching only occurring within each set [13]. A study of lichens from *Cladonia* as well as *Stereocaulon* and *Lepraria* spp. across the Canary Islands, Madeira, Sicily, and the Aeolian Islands also identified that photobiont identity was determined by both host specificity (i.e. the total number of mycobionts each photobiont formed symbioses with) and temperature, with hosts responding differently to the temperature gradient [14]. Similar patterns were observed for photobionts across these same three lichen genera in Bolivia, with lower photobiont specificity and fungal haplotype diversity in cosmopolitan host species [15].

In the genus *Umbilicaria,* three species have been extensively studied for their population turnovers along elevation gradients: *U. pustulata* and *U. phaea*, which grow in a continuum from Lower Mesomediterranean to Lower Supratemperate climate zones [16–18], and *U. hispanica,* which occurs in the Lower Supratemperate and alpine/oromediterranean biomes [19]. Part of the observed population differentiation of mycobionts in *Umbilicaria* species can be linked to environmental factors, as evidenced by abrupt genomic breaks along elevation gradients in all three species that correspond to the break in climate zones [18,20,21]. These genotypic changes between climate zones at high- and low-elevation have also been identified at functionally significant loci, including circadian- and temperature-associated genes [22], secondary metabolite genes [23] and a kinase with unknown function (24). In addition to genomic differentiation in the mycobiont, these *Umbilicaria* species also show a turnover of green algal symbionts (*Trebouxia* species) along elevation [11,18].

Although species distribution patterns for myco- and photobionts are beginning to be better described in lichens, our understanding of the specific functional consequences of these pairings is still limited. The physiological properties of lichen thalli from different environments can vary; the photosynthetic responses of *Trebouxia* species found in *U. pustulata* from high and low elevation respond differently to thallus water content, with individuals from the hotter and drier site reaching maximal photosynthesis at a lower water content than individuals from the colder and wetter site [20]. Furthermore, a study of thalli with different partner combinations has shown that photosynthetic performance can vary with both fungal and algal symbiont identity [25].

The metatranscriptional responses of lichen thalli to environmental stimuli have also begun to be dissected. In the marine cyanolichen *Lichina pygmaea,* different photobionts are more transcriptionally active depending on whether thalli are hydrated by seawater at high tide or freshwater in the form of rain at low tide [26]. In the photosymbiodeme lichen *Peltigera britannica,* where different parts of the thallus associate with either a cyanobacterial photobiont or a combination of both cyanobacterial and green algal photobionts, green algal and cyanobacterial symbionts display different thermal stress responses, while mycobionts differentially express antibiotic and growth-inhibitory genes [27]. *Evernia mesomorpha* myco- and photobiont transcriptional responses to liquid versus vapour hydration have also recently demonstrated asymmetries in how the two primary partners respond to physiological conditions [28].

Although the above studies have demonstrated how different photobionts can affect the expression profile of the lichen mycobiont and *vice versa*, it remains unclear how symbiont changes along climate gradients affect gene expression, and thus, how symbiont turnovers may be affecting the fitness landscape of lichen individuals. To explore how lichen myco- and photobiont identity affects transcriptional profiles at different climate zones, we sampled *U. pustulata* thalli from high- and low-elevation sites on Mount Limbara, Sardinia, Italy, with the high-elevation site (1250 m) being located in the Lower Supratemperate climate zone and the low-elevation site (166 m) in the Lower Mesomediterranean climate zone. To limit (but not exclude) environmental effects, we performed the experiments in a climate chamber after acclimatizing lichens over a 72 h period. To investigate additional physiological and transcriptional differences between high- and low-elevation samples, such as responses to specific environmental stimuli, we performed a commonly used experiment for testing light-responsive and circadian-associated gene expression: after dark adaptation, we applied a 20 min light pulse, which reveals early light responses in both algal and fungal partners.

Previous studies of the Mt. Limbara *U. pustulata* populations have identified strong levels of genetic variation along the climatic gradient from low to high elevation; if this genetic variation is functional in nature, we would expect these genetic patterns to be reflected in transcriptional variation between the extremes of the gradient. We therefore asked the following questions: which mycobiont and photobiont genes are differentially expressed in thalli from high- and low-elevation sites? Are previously identified genes with allele frequency differences between high- and low-elevation thalli of *U. pustulata*, such as circadian clock-associated genes, also differently expressed under acclimatized conditions? How do high- and low-elevation individuals differ in their response to environmental stimuli, such as light?

## 2. Methods

### (a) Sample collection, growth conditions and light pulse treatment

**Figure 1.**
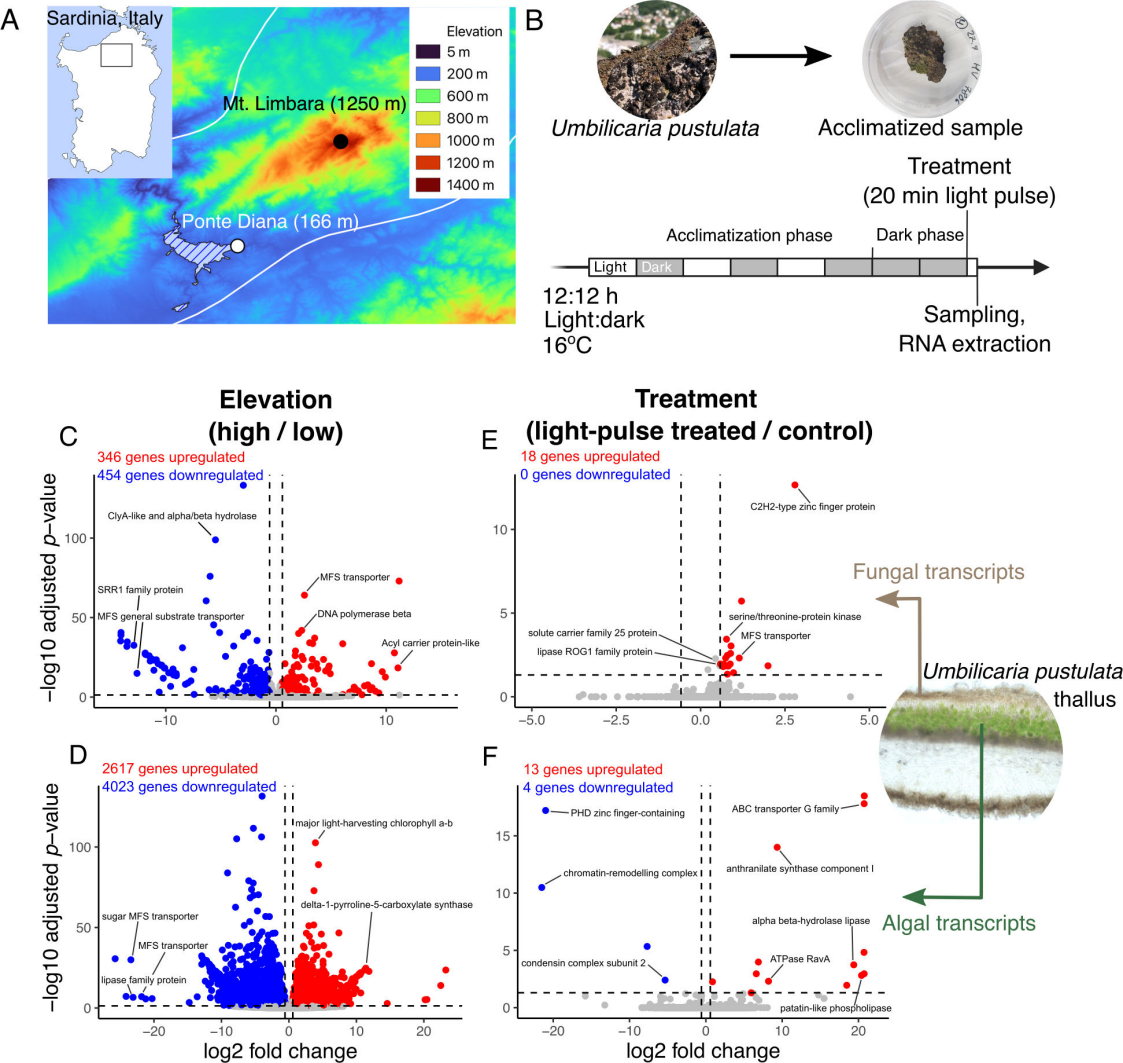
Experimental set-up and transcriptional profile of *Umbilicaria pustulata* samples taken in Sardinia, Italy. (A) Samples were harvested from a high-elevation site (Lower Supratemperate climate) and low-elevation site (Lower Mesomediterranean climate) at Mt. Limbara, Sardinia, Italy. (B) Samples were acclimatized under lab conditions for 3 days with constant humidity and temperature before a light pulse experiment was performed. Differentially expressed genes were identified between high versus low elevation (i.e. upregulated genes are overexpressed in high-elevation samples relative to low-elevation samples) in fungal (C) and algal (D) transcripts, and between light pulse-treated and control samples in fungal (E) and algal (F) transcripts. Horizontal dotted line denotes −log(0.05) and vertical dotted lines denote +/−1.5-fold change in expression.

In order to determine the extent of transcriptional variation for both myco- and photobionts in different climate zones, we collected *U. pustulata* thalli for RNA-seq analysis at the two most extreme points along the climatic gradient: a low-elevation site at Ponte Diana, adjacent to Coghinas Lake (166 m, 40°45'26.3333"N, 9°04'41.9322"E, ‘Ponte Diana’ in figure 1A), and a high-elevation site near the peak of Mount Limbara (1250 m, 40°51'24.6341"N, 9°10'06.8779"E, ‘Mt. Limbara’ in figure 1A). A detailed categorization of the bioclimatic conditions at the sampling sites can be taken from [29]. The map in this publication shows that the low elevation site by Coghinas lake is in the overall bioclimatic category ‘Mediterranean Pluviseasonal-Oceanic’, more precisely ‘Lower Mesomediterranean (isobioclimate 17)’, while the high elevation site is in the overall category ‘Temperate Oceanic’, more precisely ‘Lower Supratemperate (isobioclimate 40)’. We thus characterize the high-elevation site as ‘Lower Supratemperate’ and the low-elevation site as ‘Lower Mesomediterranean.’ Thalli were collected from sun-exposed granitic rocks on 29 March 2022, air-dried and transported to a growth chamber in Frankfurt am Main, Germany.

We assigned *Trebouxia* species following [30] based on internal transcribed spacer (ITS) rDNA sequences. The low elevation thalli contained taxon S19 (reference GB accession MH299167), which we have previously also referred to as operational taxonomic unit 3 (OTU3) [31], OTU_alga_5 [11] and haplotype 04 (HA04) [21]. The high-elevation thalli contained a taxon in the S clade of *Trebouxia*, which according to [30] has no number assignment yet. We refer to this taxon as OTU2 (as in [31]; GB accession MH299133). We selected four low-elevation thalli containing S19, and four high-elevation thalli containing OTU2.

In order to minimize some of the transcriptional variation associated with environmental conditions at each site during sampling, *U. pustulata* thalli were rehydrated and acclimatized over 72 h in a climate chamber before being subjected to a 32 h dark adapted phase followed by a 20 min light pulse treatment and subsequent RNA-seq analysis.

Growth conditions and the light pulse treatment were performed as in [32]. Briefly, dried *U. pustulata* individuals were separated into two thallus pieces, one for the dark-adapted (DD) and one for the light pulse sample (LL). Thallus pieces were placed in closed 6 cm Petri dishes and rehydrated by applying 0.5 ml dH_2_O to blotting paper and allowing lichen thalli to rehydrate through both water vapour and partial direct contact with the blotting paper, while acclimating to laboratory conditions over 3 days with 12 h light/12 h dark cycles (60 μmol photons m^−2^ s^−1^) at 16°C. Thalli were kept hydrated with dH_2_O applied to the filter paper in contact with each thallus piece once during each day. After 3 days, samples were then exposed to an additional 24 h of constant darkness, before light pulse-treated samples were exposed to a 20 min light pulse (60 μmol photons m^−2^ s^−1^) at 08:00 (circadian time 0, subjective dawn) and both control (no light pulse) and light pulse-treated samples were harvested immediately into liquid nitrogen and stored at −80°C before RNA extraction.

### (b) RNA extraction, library construction and RNA sequencing

We sampled control (no light pulse) and light pulse-treated thallus pieces (*ca* 150 mg each), ground them under liquid nitrogen using a mortar and pestle, and extracted RNA with TRI Reagent (Zymo Research Europe GmbH, Freiburg, DE) according to the manufacturer’s instructions. The extracted RNA was then sent to Novogene (Cambridge, UK) for library construction, quality control (QC) and 150 bp paired-end sequencing with NovaSeq. All sequence information can be found on the European Nucleotide Archive (ENA) under project accession number PRJEB72275.

### (c) Differential gene expression analysis

Differential gene expression (DGE) analysis was done by adapting the pipeline established by Mistry *et al.* [33]. The most recently annotated genome of *U. pustulata* published in [23] was used for fungal read mapping. For algal read mapping, *Trebouxia* sp. scaffolds derived from sequencing pure algal cultures were used. Because a genome of the high-elevation OTU2 is currently not available, reads from both the high-elevation OTU2-containing and the low-elevation S19-containing thalli were mapped to a third S-clade *Trebouxia* species, S12 C0005, that has recently been sequenced with high Benchmarking Universal Single-Copy Orthologs (BUSCO) completeness and low number of scaffolds [34].

After QC and trimming of raw reads using *FastQC* v.0.11.9 [35] and *Trimmomatic* v.0.39 [36], indexing (*salmon index*) and quantification of RNA transcripts was performed using *Salmon* v.0.13.1 [37]. QC of the mapped sequences was done using *HISAT2* v.2.2.1 [38] and *SAMtools* v.1.17 [39] to align reads to *U. pustulata* and S12 C0005 *Trebouxia* genome scaffolds, followed by *QualiMap 2* v.2.3 [40]. All QC information was subsequently aggregated and analysed using *MultiQC* v.1.14. DGE analysis was conducted using *DESeq2* v.1.38.3 [41] in *R*. From these two datasets (fungal and algal reads), a total of 8772 fungal and 17 629 algal transcripts were quantified from the metatranscriptomic dataset.

Gene IDs were confirmed, when possible, by running protein sequences from genes of interest (e.g. identified by DESeq2 analysis for light treatment and elevation, electronic supplementary material, tables S1–S4) against InterPro protein Basic Local Alignment Search Tool (BLAST), with proteins that could not be identified (%ID below 40%) otherwise labelled as ‘hypothetical protein’. Conserved domains were identified, when possible, by running protein sequences against National Center for Biotechnology Information (NCBI) Conserved Domains Search (CDD v.3.21).

### (d) Weighted gene co-expression network analysis

The identification of co-expressed genes in parallel to DESeq2 analyses was done using Weighted Gene Co-expression Network Analysis (WGCNA) [42] in R. Briefly, after normalization of read counts for all genes, we determined the soft threshold level for the ‘power’ parameter using the *pickSoftThreshold* function by calculating a measure of the model fit, the signed *R*^2^, above 0.80. We subsequently ran *blockwiseModules* (power = 14 for fungal reads, 9 for algal reads; TOMType = ‘signed’; maxBlockSize = 20000; randomSeed = 1234*).* We used limma v.3.54.2 [43] in order to determine if any of the identified modules’ eigengenes were significantly correlated to treatment or site.

## 3. Results

### (a) *U. pustulata* fungal and algal transcriptomic fractions demonstrate elevation-associated and light pulse-responsive transcriptional variation

After mapping to *U. pustulata* Ascomycota scaffolds (hereafter, fungal transcripts) and *Trebouxia* S12 C0005 scaffolds (hereafter, algal transcripts), we performed differential expression analyses using DESeq2. A large number of fungal (800, figure 1C and electronic supplementary material, table S3) and algal (6640, figure 1D and electronic supplementary material, table S4) transcripts were differentially expressed between high- and low-elevation populations. A small number of fungal (18, figure 1E and electronic supplementary material, table S1) and algal (17, figure 1F and electronic supplementary material, table S2) transcripts were differentially expressed by the 20 min light pulse (DESeq2 adjusted *p*‐value < 0.05, fold change > 1.5). The 800 high-/low-elevation fungal DEGs were over-represented in carbohydrate and vitamin/thiamine (vitamin B1) metabolic processes (electronic supplementary material, figure S1A), while the 6640 high-/low-elevation algal DEGs were over-represented in a variety of secondary metabolite biosynthesis processes, including terpenoid and isoprenoid biosynthetic processes (electronic supplementary material, figure S1D).

### (b) Fungal and algal genes show different high- and low-elevation co-expression patterns

**Figure 2.**
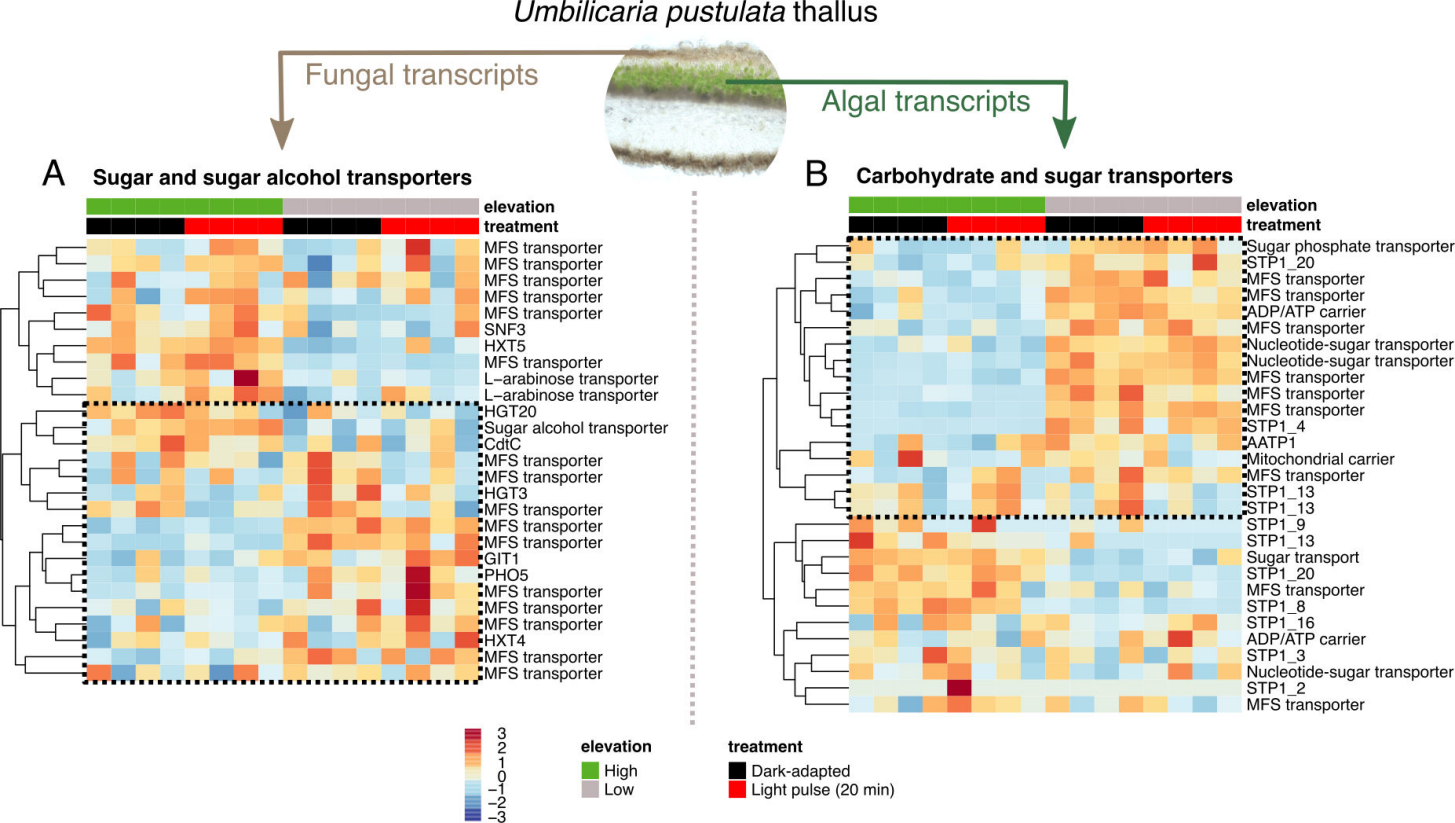
Fungal and algal sugar transporters show similar high- and low-elevation co-expression patterns. (A) Fungal reads. (B) Algal reads. Dashed boxes indicate sugar transporters that are downregulated in high-elevation individuals. A light pulse treatment has no effect on fungal and algal sugar, sugar alcohol and carbohydrate-associated gene expression.

Previous work has identified large differences in genes linked to carbohydrate and sugar transport among lichen-forming fungi [44]. Here, we compared the expression levels of sugar, sugar alcohol and carbohydrate transporters in both the fungal and algal fractions of the transcriptome. Sugar transporters were among the most differentially expressed genes in both the fungal and algal transcripts. We identified a set of 27 sugar and sugar alcohol transporters in the fungal transcripts (figure 2A) and a set of 29 carbohydrate and sugar transporters in the algal transcripts (figure 2B). For both the fungal and algal transcripts, a sharp division between transporters that were overexpressed in high- versus low-elevation fungal genotypes and algal species were observed, with 17/27 (63%) fungal and 17/29 (59%) algal sugar transporters forming a cluster downregulated in the high-elevation individuals, while 10/17 (37%) fungal and 12/29 (41%) algal sugar transporters forming a cluster downregulated in the low-elevation individuals.

### (c) Circadian- and temperature-associated genes show elevation-specific co-expression, but not light pulse-dependent patterns

**Figure 3.**
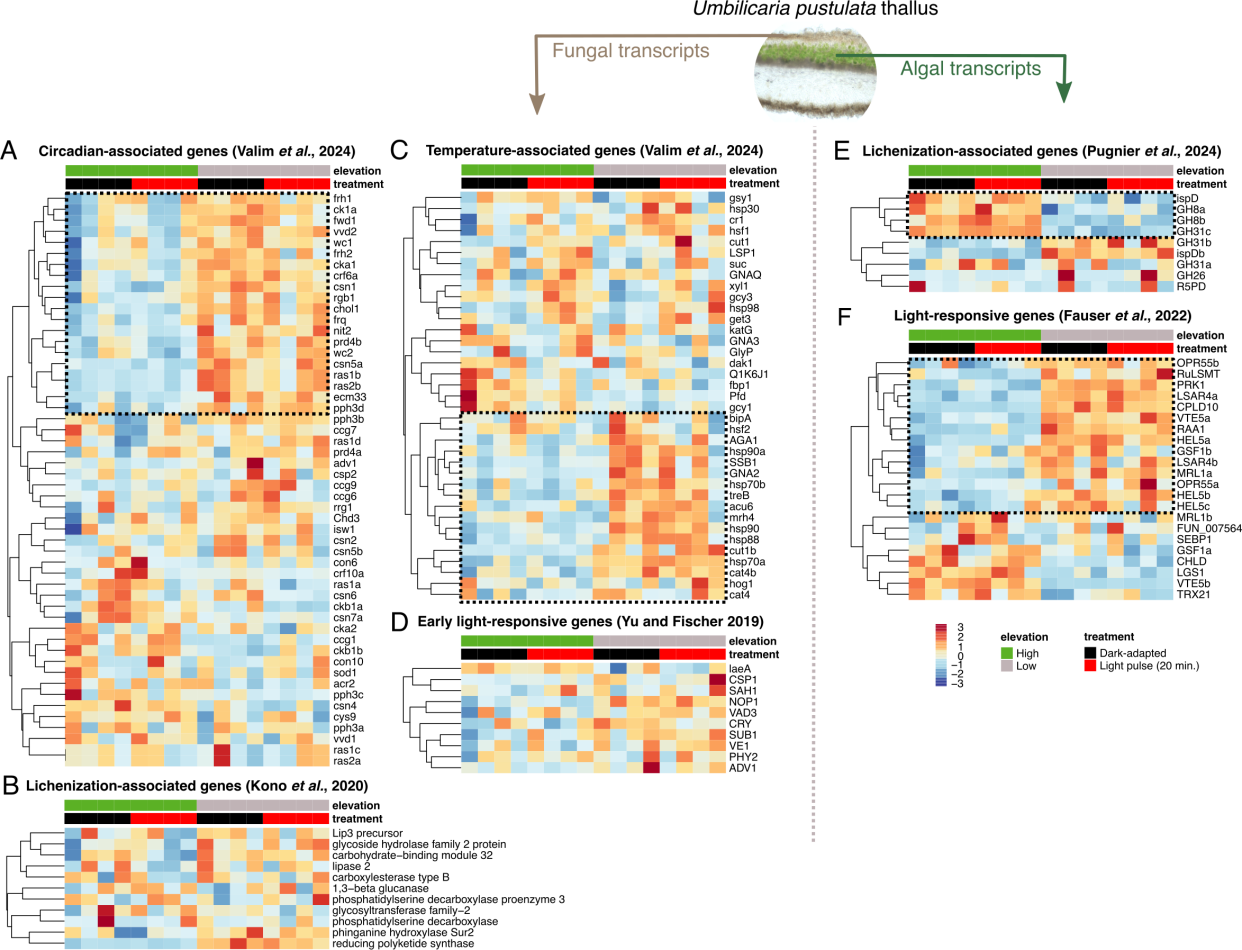
Circadian- and temperature-associated as well as light-responsive genes show different patterns at high- and low-elevation sites for fungal and algal reads. (A) Circadian-associated fungal genes identified in Valim *et al*. [45]. (B) Lichenization-associated fungal genes identified in Kono *et al*. [46]. (C) Temperature-associated fungal genes identified in Valim *et al*. [45]. (D) Early light-responsive fungal genes derived from Yu & Fischer [47]. (E) Lichenization-associated algal genes identified in Puginier *et al*. [48]. (F) Light-responsive algal genes identified in Fauser *et al*. [49]. Dashed boxes indicate genes that are downregulated in high-elevation individuals.

We previously identified a set of 50 circadian- and 37 temperature-associated fungal genes with allelic variation in high- and low-elevation populations of *U. pustulata* across several elevation gradients, including Mt. Limbara, Sardinia [22]. Here, we analysed the expression profile of these gene sets and found a cluster of genes in the circadian-associated (20/52, 38%, figure 3A) and temperature-associated (17/37, 46%, figure 3C) gene sets that were downregulated in the high-elevation fungal genotype.

We analysed the expression profile of two additional gene sets of interest, lichenization-associated and light-responsive genes, in both the fungal and algal reads. The algal ‘lichenization-associated gene set’ was derived from a phylogenomic analysis of the evolution of lichenization by [48] and for the fungal reads from a metatranscriptomic study of lichen symbiosis resynthesis experiments by [46]. The algal light-responsive gene set was derived from homologues of light-sensitive genes characterized in *Chlamydomonas reinhardtii* [49] and for the fungal reads from well-known early light signalling genes in *N. crassa* (reviewed in [47]). Both gene sets demonstrated some algal species-specific clustering: 4/9 of the algal lichenization-associated genes were downregulated in the low-elevation species (figure 3E), while 14/22 (64%, figure 3F) of the light-responsive genes were downregulated in the high-elevation species. However, neither the lichenization-associated (figure 3B) nor the early light-responsive fungal genes (figure 3D) demonstrated clustering in a genotype- or treatment-specific manner.

### (d) Weighted gene co-expression module analysis identifies fungal- and algal-specific light pulse-responsive modules

In order to further investigate how mycobiont expression patterns were affected by the light treatment or the identity of both the fungal genotype and algal symbiont species, we performed a weighted gene co-expression network analysis (WGCNA) to identify modules of co-expressed genes. We identified 56 modules of co-expressed genes for the fungal transcripts (electronic supplementary material, figure S2A–C) and 9 modules for the algal transcripts (electronic supplementary material, figure S2D–F). 27/56 (48%) of the fungal gene co-expression modules were significantly correlated (adjusted *p*-value < 0.05) with high- versus low-elevation genotype identity, comprising 3868/8772 (44%) of all fungal transcripts quantified (electronic supplementary material, table S5); 2/9 of the algal gene co-expression modules were significantly correlated (adjusted *p-*value<0.05) with high- versus low-elevation species identity, comprising 10 384/17 629 (59%) of all algal transcripts quantified (electronic supplementary material, table S6). To ascertain the overlap between the WGCNA and DESeq2 approaches, we compared transcripts in the largest co-expression modules for both the fungal and algal transcripts with the DESeq2 results for high- versus low-elevation genotype/species. For the fungal genes, 554 transcripts overlapped between the WGCNA module 1 and DESeq2 elevation results, accounting for 45% and 69% of the total genes, respectively (electronic supplementary material, figure S3A). For the algal genes, 6051 transcripts overlapped between the WGCNA module 1 and DESeq2 elevation results, accounting for 66% and 91% of the total genes, respectively (electronic supplementary material, figure S4A).

**Figure 4.**
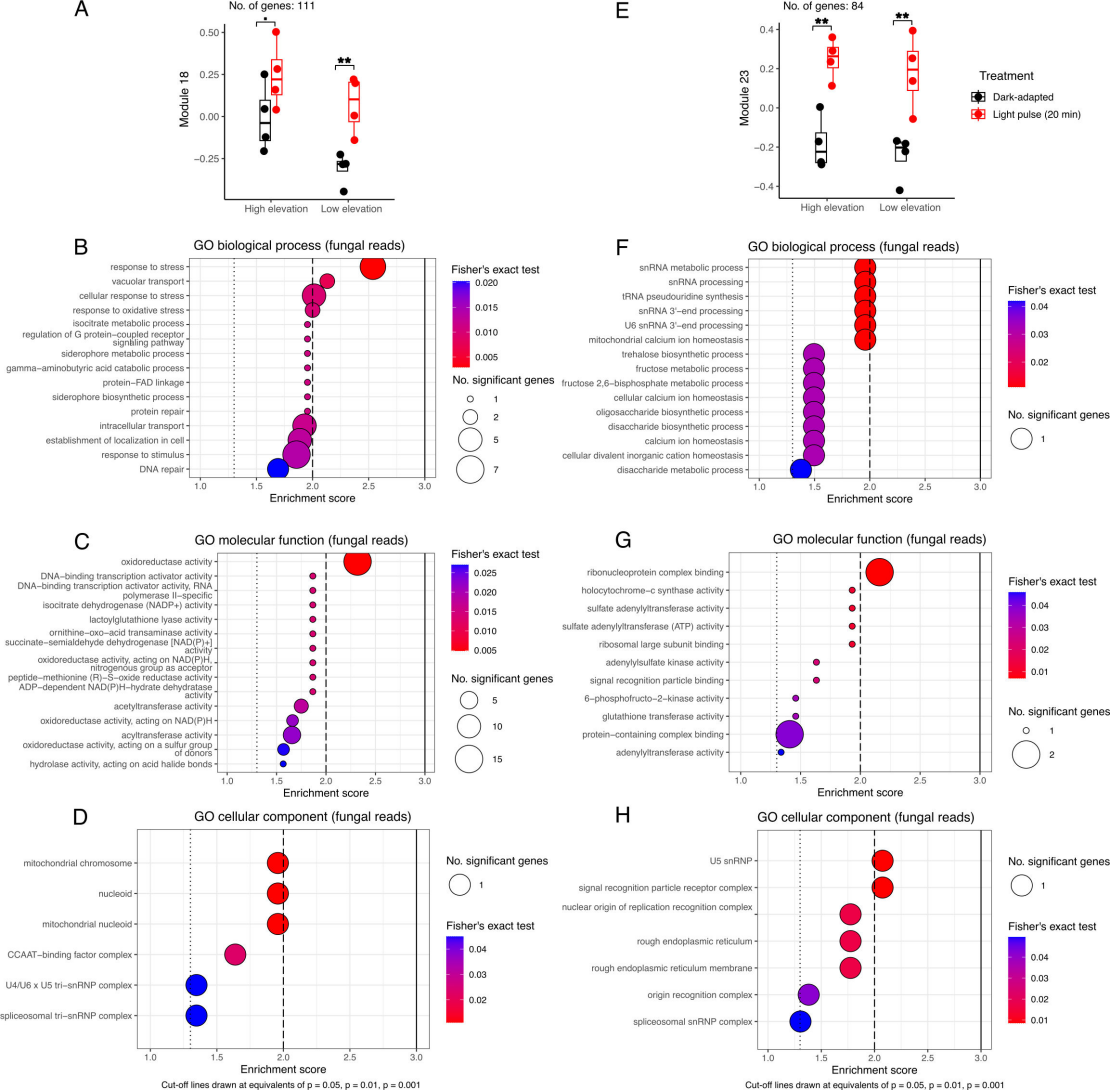
Two co-expression modules in lichen fungal reads characterize early light pulse-dependent responses. Panels (A–D) correspond to fungal module 18 (111 genes). Panels (E–H) correspond to fungal module 23 (84 genes). (A,E). Plotting of eigengene values. (B, F). Gene set enrichment analysis (GSEA) of biological process gene ontology (GO) terms. (C, G). GSEA of molecular function GO terms. (D, H). GSEA of cellular component GO terms. Dashed and solid lines in GSEA plots denote *p* = 0.05, *p* = 0.01 and *p* = 0.001 for Fisher’s exact test results for each GO term. NAD(P)H: nicotinamide adenine dinucleotide phosphate; snRNA: small nuclear RNAs; snRNP: small nuclear ribonucleoproteins; FAD: flavin adenine dinucleotide.

We identified two modules in the fungal transcripts that were differentially expressed by the 20 min light pulse treatment. Fungal module 18 (111 genes, figure 4A), was enriched in stress response and transport biological processes (figure 4B) as well as molecular functions associated with the activation of core metabolism, such as oxidoreductase activity, nicotinamide adenine dinucleotide phosphate (NADPH/NADP^+^) activity and transcription activation (figure 4C). Cellular localization results for fungal module 18 point to transcriptional activation at both the nucleus and mitochondrial nucleoid (figure 4D). Fungal module 23 (84 genes, figure 4E), was enriched in a variety of sugar biosynthetic processes and transcriptional activation (figure 4F), as well as a variety of molecular functions involved in transcriptional activation and ribosomal activation (figure 4G). Cellular localization results for fungal module 23 point to activation of the transcriptional/translational machinery and protein mobilization in the rough endoplasmic reticulum (figure 4H).

**Figure 5.**
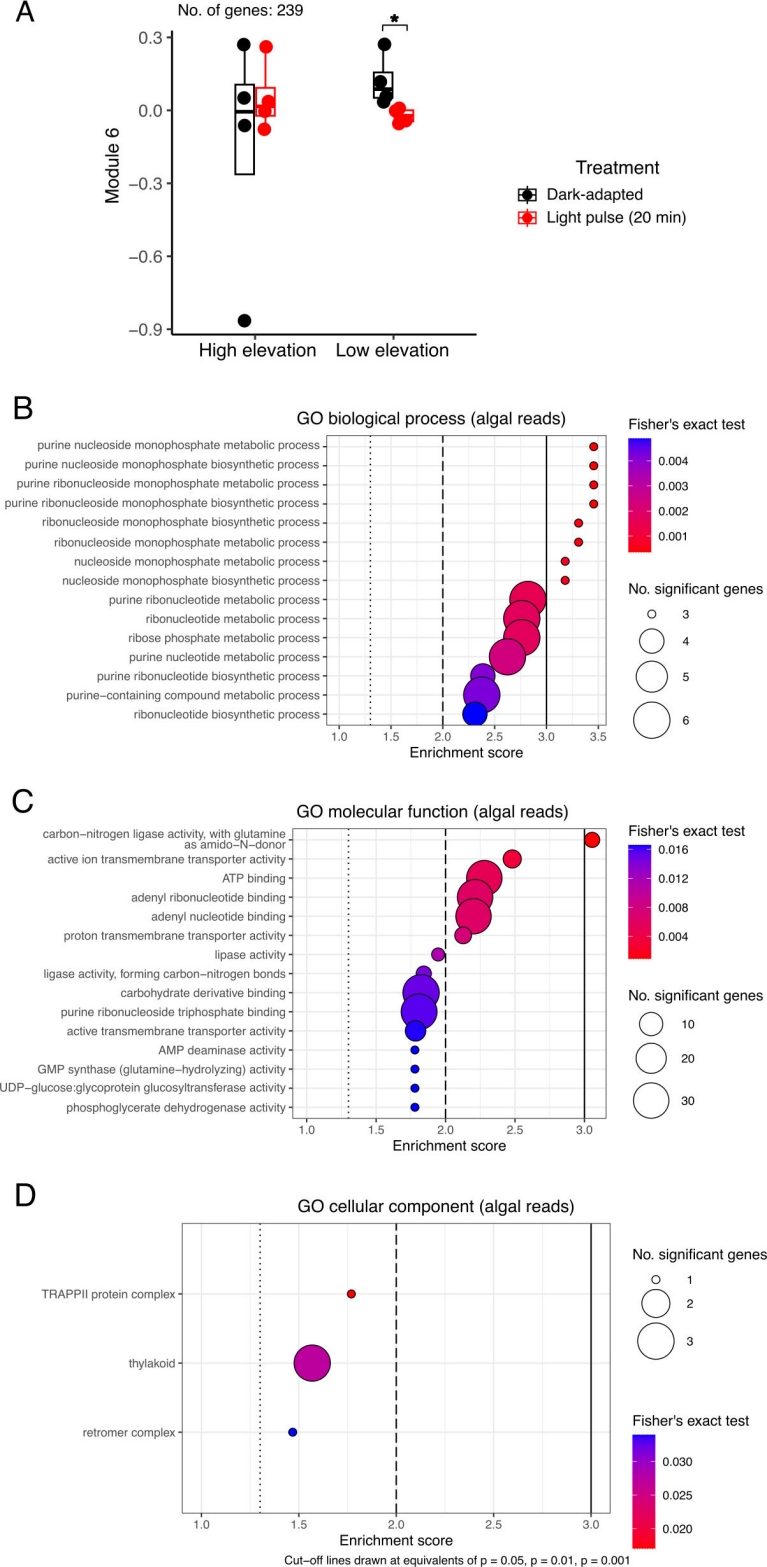
A co-expression module in lichen algal reads reveals species-specific early light pulse-dependent responses. (A) Plotting of eigengene values for genes in algal module 6 (239 genes). (B) Gene set enrichment analysis (GSEA) of biological process gene ontology (GO) terms. (C) GSEA of molecular function GO terms. (D) GSEA of cellular component GO terms. Dashed and solid lines in GSEA plots denote *p* = 0.05, *p* = 0.01 and *p* = 0.001 for Fisher’s exact test results for each GO term. ATP: adenosine triphosphate; AMP: adenosine monophosphate; GMP: guanosine monophosphate; UDP: uridine diphosphate; TRAPP: TRAnsport Protein Particle

We identified one module in the algal transcripts that was downregulated by the 20 min light pulse in the low-elevation individuals only. Algal module 6 (239 genes, figure 5A), was enriched in ribonucleotide and nucleotide biosynthetic processes (figure 5B), transmembrane transporter and nucleotide binding molecular functions (figure 5C) that were partly localized to the thylakoids and TRAPPII complex in the protein transport chain (figure 5D).

## 4. Discussion

### (a) Both fungal and algal symbiont expression profiles vary between high- and low-elevation populations of *U. pustulata*

Previous work in *U. pustulata* and other Umbilicariacae has painted a clear picture of genotypic variation along elevation gradients in both fungal and algal symbionts [20–22]. However, it has remained unclear whether functional differences exist at the molecular level between lichen individuals from high elevations (with specific symbiont pairings) versus individuals from low elevations (with different, specific symbiont pairings). Here, we report high variation in constitutive gene expression profiles between high- and low-elevation *U. pustulata* thalli. Constitutive variation in gene expression was much higher between the two algal species, with 6640 DEGs (38% of all algal genes, figure 1C), compared with only 800 DEGs in the two fungal genotypes (9% of all fungal genes, figure 1D).

Lichenizing fungi and algae are both known to produce a host of compounds, including phytohormones, that regulate stress responses and may be used for inter-symbiont signalling. Jasmonic acid (JA) and especially indole-3-acetic acid (IAA), in the auxin class, are known to be produced by lichenizing fungi in culture, with IAA being known to increase *Trebouxia* water content and fresh mass in biologically relevant concentrations [50]. Various classic phytohormones, including IAA and abscisic acid (ABA), are released by several microalgae, with JA and gibberellin GA3 also being exuded extracellularly by *Trebouxia* sp. [51]. These phytohormones are known to mediate fungal [52] and plant stress responses [53], and may play an additional role in mediating signalling between fungal and algal symbionts. Thiamine/biotin (vitamin B1), which was differentially expressed in the fungal reads, is also known to play a role in oxidative stress responses in fungi [54,55]. In addition, Major Facilitator Superfamily (MFS) transporters, a ubiquitous class of small-molecule transporters [56] that were differentially expressed in both the fungal and algal reads (figure 1C–F), may also point to an interplay in small molecules for signalling back and forth between the fungal and algal symbionts.

The classic view of the lichen symbiosis is that the mycobiont benefits by receiving a steady carbon source from the photobiont, both for energy and increased desiccation tolerance, and provides protection from environmental stresses to the photobiont in return [2]. Changes in lichen symbiont identity are probably adaptive along climate gradients, but most lichen studies have focused on larger temperate, boreal and alpine lichens in North America and Europe, limiting our knowledge of these dynamics at the physiological and metagenomic level [57]. Here, we show that algal symbionts in particular are highly variable in their transcriptional capabilities at both extremes of a climate gradient. Previous work has suggested that generalist lichen photobionts are more broadly plastic in their photosynthetic responses [58]. Alongside photobiont identity, this plasticity appears to be strongly influenced by the mycobiont’s protective abilities, e.g. how much arabitol it can provide to photobionts and its effect on light stress under desiccation [59]. Our findings provide evidence that there is likely local adaptation in species with large ranges such as *U. pustulata*.

Although we observe high variation between high- and low-elevation sites for both the myco- and photobiont, our understanding of whether this is driven by the genotypic or climatic/ontogenetic variation could not be resolved. Because the sites, climate and elevation zones and genotypes are clustered together (e.g. S19 was only found at low-elevation sites and OTU2 was only found at high-elevation sites), this conflation cannot be resolved using our experimental design. Future studies should compare high- and low-elevation sites (or sites in different climate zones) that contain the same myco- and/or photobiont lineages, in order to disentangle the genotypic and climatic influences.

### (b) *U. pustulata* light-responsive and circadian clock-associated genes vary between high- and low-elevation sites

We identified a set of circadian-associated (figure 3A) and temperature-associated (figure 3C) genes in the fungal symbiont that were constitutively downregulated in the high-elevation genotype, while a set of light-responsive genes (figure 3F) in the algal symbiont was similarly downregulated in the high-elevation genotype. Curiously, none of these gene sets displayed clear evidence of induction or downregulation by the 20 min light pulse. The set of fungal circadian genes that was downregulated in the high-elevation genotype included the core circadian clock components *frequency* (frq), *FRQ-interacting RNA helicase* (frh), *white collar 1* (wc-1) and *white collar 2* (wc-2). We have previously analysed the light pulse activation of frq in *U. pustulata* (Lecanoromycetes) and *Dermatocarpon miniatum* (Eurotiomycetes); looking at this and the other core circadian clock components more closely, there is a non-significant light pulse effect for frq in the high-elevation genotype, while the low-elevation genotype displays constitutively higher levels of frq expression (electronic supplementary material, figure S5A). It may be that the 20 min light pulse is not sufficient to identify frq light-dependent activation across *U. pustulata* genotypes, and that lichen-forming fungi display slower transcriptional response times than other fungi [60,61]. Alternatively, the light pulse intensity chosen (60 μmol photons m^−2^ s^−1^) may have been too low for a reliable induction of light-responsive genes in lichens.

Both core and peripheral circadian clock genes, like those in the circadian-associated gene set from [45] (figure 3A), vary along climatic gradients in other species. Two classic examples of functional variation in core circadian clock genes, both in *Drosophila melanogaster*, are the variation in the length of threonine–glycine pair repeats in the gene *period* and the frequency of two variants of the gene *timeless,* both of which vary along a latitudinal gradient [62]. Several examples of functional variation in peripheral circadian clock-associated genes exist e.g. in plants, where many are flowering time-associated. One example is a latitudinal gradient in the allele frequency of *COR28*, a flowering time- and freezing tolerance-associated gene in *Arabidopsis thaliana* [63]. Further transcriptional profiling along the Mt. Limbara elevational gradient, rather than just at the extremes, may inform whether some transcripts form a clinal pattern similar to those described above. This would provide important functional evidence for the adaptive value of the circadian clock in lichen mycobionts beyond the level of genomic inference [22].

The individual mycobiont lineages and photobiont species identified at Mt. Limbara have been identified across the European gradient for *U. pustulata*, although this investigation has only been done at the single-marker level [64,65]. Future work should explore what fraction, if any, of the alleles for the genes identified in [22,45] in this work are shared between high-elevation Mediterranean sites such as at Mt. Limbara and cold temperate sites more north of the Alps in Europe. This comparison would also better inform us about which genes vary along a latitudinal cline and are thus involved in photoperiodic- and circadian-associated adaptive responses.

### (c) Lichenization-associated and light-sensitive algal genes, but not fungal genes, display strong partitioning between high- and low-elevation thalli

We analysed the expression profile of a ‘lichenization-associated gene set’ derived from a phylogenomic analysis of the evolution of lichenization in algae by [48], finding that 4/9 of the lichenization-associated genes were downregulated in low-elevation individuals, while the other 5/9 genes were downregulated in high-elevation individuals (figure 3E). Similarly, 14/22 of the algal light-responsive genes (64%, figure 3F) were downregulated in high-elevation individuals. By contrast, no clear patterns emerged for the fungal lichenization-associated genes identified by [46] (figure 3B) or for early fungal light-signalling genes (figure 3D).

These stronger elevation-dependent signals for the algal but not the fungal partner may point to differences in the degree of genetic variation between high- and low-elevation thalli: the fungal partner is highly differentiated along the genome, but is considered the same species, while the algal partners at high and low elevation are considered different species [18]. In addition, the stronger signals for the algal symbionts may demonstrate variable selection pressures for the algae. This has been hypothesized to occur in lichens due to the nature of the symbiosis: lichen-forming fungi sporulate and recruit local algae at the site of germination when undergoing sexual reproduction, while the algae in the thalli only undergo asexual reproduction and have their growth rates curtailed in symbiosis [66]. This latter possibility would be unexpected in *U. pustulata,* the lichen under study here; however, this species is known to only undergo asexual reproduction, without sporulation [16], which is confirmed by comparisons to a sexually reproducing species in the same genus, *U. phaea* [22,45]. More systematic evidence of functional and genomic variation in the algal and fungal fractions of other sexually and exclusively asexually reproducing lichens along climate gradients, like those of *U. phaea* and *U. pustulata,* will need to be gathered to better understand any potential coevolutionary relationship between lichen symbionts.

### (d) Sugar transport genes demonstrate climate-specific patterns in both fungal and algal reads

Changes in carbohydrate metabolism and transport constitute important metabolic adaptations to the environment in plants [67]. Sugar transport has been linked to drought adaptation, heat stress and cold stress (reviewed in [67,68]). Our results suggest that different sugar transport mechanisms operate in high- and low-elevation individuals in both symbionts, perhaps indicating adaptation to different types of abiotic stress. Low-elevation individuals experience extended periods of drought over the Mediterranean summer, putatively using sugars and polyols for osmoprotection and antioxidant defence, like plants and other lichens [69,70]. High-elevation individuals experience freezing in winter, potentially using sugars as cryoprotectants [67].

Differences in sugar transport regulation in the fungal partner may be linked to interaction with different *Trebouxia* species. Plant pathogens can upregulate sugar transporters in host plants to improve their own supply of nutrients, and different pathogens may upregulate different transporters [71]. The biosynthesis and transport of the sugar trehalose, for example, is a common target in studies of fungal pathogenicity in plants [72–74]. Thus, the specific molecular mechanisms by which the fungus in the lichen symbiosis acquires carbohydrates from the algal symbiont may depend on the specific biotic interaction and taxonomic identity of the algal partner.

We observed constitutive differences between high- and low-elevation genotypes in fungal and algal sugar transporters, but no activation by the 20 min light pulse in either (figure 2A,B). This is consistent with previous work in *E. mesomorpha,* which has similarly demonstrated that sugar transporter expression is not drastically affected by other treatments, like shifts from liquid versus vapour hydration [28]. We observed that fungal and algal sugar transporters fall broadly into two co-expressed clusters in the high- or the low-elevation fungal and algal reads. These results were also consistent with the DESeq2 analysis, where several MFS transporters were identified as among the most DEGs in both the fungal and algal fractions of the metatranscriptome (figure 1D,E). These genes, part of the Major Facilitator Superfamily (MFS), are well-conserved membrane transporters of sugars and sugar alcohols, among other compounds [56].

It remains to be studied whether the different sugar transporters being expressed in high- versus low-elevation fungal and algal symbionts affect sugar transport dynamics (e.g. different rates of sugar or sugar alcohol transport from algal to fungal partners and *vice versa*), or whether they might lead to the transport of different sugars or sugar alcohols. Further work should quantify sugar and sugar alcohol concentrations in high- and low-elevation genotypes of *U. pustulata* under different conditions, both in the field and after acclimatization in the laboratory, to better understand how these may vary along elevation gradients. Fungal-derived sugar alcohols like arabitol have been previously demonstrated to improve desiccation tolerance in lichen-forming algae [59], which may imply that variable sugar transport may be adaptive.

### (e) Early light-responsive genes in fungal and algal symbionts point to a coordinated assimilation response in low- but not high-elevation individuals

Only a few DESeq2-identified genes in the dataset responded to the 20 min light pulse (18 fungal and 17 algal genes, adjusted *p*‐value < 0.05, fold change > 1.5). Of these early light-responsive genes, transporter- and lipase-associated biosynthetic genes were identified in both fungal and algal symbionts, pointing to a coordinated early response to the light pulse. Interestingly, the WGCNA co-expression analysis identified two additional sets of light-responsive genes in the fungal reads, one with stress response and solute transport-associated genes over-represented (111 genes, figure 4A–D) and another with a sugar biosynthetic process over-represented (84 genes, figure 4E–H). A single module (239 genes, figure 5A–D) of algal light-responsive genes was identified in the low-elevation individuals, over-represented in transmembrane transporter activity in the thylakoid, perhaps denoting an additional set of early light-activated photosynthesis-associated genes.

Lichens at high- versus low-elevation sites are under variable selective pressures, with high-elevation thalli at the top of Mt. Limbara experiencing temperatures below zero during winter and higher mean annual precipitation (around 1400 mm [32]). Low-elevation thalli experience higher temperatures (above zero throughout the year) and much lower mean annual precipitation (less than 300 mm on average [32]). Variation in responsiveness to the light pulse between high- and low-elevation fungal and algal symbionts may be tied to variable stress regimes experienced by each symbiont at each site. Light signalling in plants is known to affect a variety of abiotic stress responses: the red light photoreceptors, phytochromes, are known to regulate germination responses to differing temperatures [75], and light signalling pathways are an important component of plant drought responses [76]. In the fungal pathogen *Alternaria alternata,* the blue light photoreceptor LreA affects gene expression in a temperature-dependent way, implying that temperature responses may mimic or co-opt photoresponses in a similar way in fungi [77].

In symbiotic associations, the partners may show either closely coordinated or unsynchronized transcriptional responses to external stimuli. Examples of coordinated responses include in the cereal weevil *Sitophilus oryzae* and its endosymbiont *Sodalis pierantonius,* where a variety of responses are co-regulated between the host and endosymbiont throughout *S. oryzae*’s life cycle, pointing to a coordinated transcription between them [78]. In the pea aphid *Acyrthosiphon pisum* and its endosymbiont *Buchnera aphidicola,* evidence of co-biosynthesis between them in host tissues inhabited by the endosymbiont is consistent with the hypothesis that *B. aphidicola* uses host-derived polyols as a food source [79].

Uncoordinated responses have been observed in the coral *Oculina arbuscula* and its endosymbiont *Breviolum psygmophilum* to temperature stress, where symbiosis status strongly affected the response of the symbiont, but not of the host, while aposymbiotic *B. psygmophilum* displayed greater temperature stress responses than *B. psygmophilum* in symbiosis, pointing to a buffering effect of the symbiont by the coral host [80]. Recent evidence from lichens also points to unsynchronized transcriptional responses between myco- and photobionts to external stimuli: in *Evernia mesomorpha,* where only myco- but not photobionts respond to different hydration sources [28], and in the marine lichen *Lichina pygmaea* , where different symbiotic cyanobacteria become more transcriptionally active under either fresh- or saltwater hydration regimes [26].

Here, we observe an asynchronous response to a light pulse for the high-elevation individuals. However, we also observe a more synchronized early light response between fungal and algal symbionts for low-elevation individuals. Together, these provide the first evidence of metatranscriptional responses to a stimulus in *Trebouxia-*containing lichens from the same species, but different climate zones.

## Data Availability

The raw data for this study have been deposited in the European Nucleotide Archive (ENA) at European Molecular Biology Laboratory European Bioinformatics Institute (EMBL-EBI) under accession number PRJEB72275. All scripts, fungal/algal mapped reads and necessary data files for reconstructing the datasets in this study are available on Dryad [81]. Supplementary material is available online [82].
